# Genome Sequencing of Idiopathic Pulmonary Fibrosis in Conjunction with a Medical School Human Anatomy Course

**DOI:** 10.1371/journal.pone.0106744

**Published:** 2014-09-05

**Authors:** Akash Kumar, Max Dougherty, Gregory M. Findlay, Madeleine Geisheker, Jason Klein, John Lazar, Heather Machkovech, Jesse Resnick, Rebecca Resnick, Alexander I. Salter, Faezeh Talebi-Liasi, Christopher Arakawa, Jacob Baudin, Andrew Bogaard, Rebecca Salesky, Qian Zhou, Kelly Smith, John I. Clark, Jay Shendure, Marshall S. Horwitz

**Affiliations:** 1 University of Washington School of Medicine, Seattle, Washington, United States of America; 2 Medical Scientist Training Program (MSTP), University of Washington, Seattle, Washington, United States of America; 3 Department of Genome Sciences, University of Washington, Seattle, Washington, United States of America; 4 Department of Pathology, University of Washington, Seattle, Washington, United States of America; 5 Department of Biological Structure, University of Washington, Seattle, Washington, United States of America; Medical School Hannover, Germany

## Abstract

Even in cases where there is no obvious family history of disease, genome sequencing may contribute to clinical diagnosis and management. Clinical application of the genome has not yet become routine, however, in part because physicians are still learning how best to utilize such information. As an educational research exercise performed in conjunction with our medical school human anatomy course, we explored the potential utility of determining the whole genome sequence of a patient who had died following a clinical diagnosis of idiopathic pulmonary fibrosis (IPF). Medical students performed dissection and whole genome sequencing of the cadaver. Gross and microscopic findings were more consistent with the fibrosing variant of nonspecific interstitial pneumonia (NSIP), as opposed to IPF *per se*. Variants in genes causing Mendelian disorders predisposing to IPF were not detected. However, whole genome sequencing identified several common variants associated with IPF, including a single nucleotide polymorphism (SNP), rs35705950, located in the promoter region of the gene encoding mucin glycoprotein MUC5B. The *MUC5B* promoter polymorphism was recently found to markedly elevate risk for IPF, though a particular association with NSIP has not been previously reported, nor has its contribution to disease risk previously been evaluated in the genome-wide context of all genetic variants. We did not identify additional predicted functional variants in a region of linkage disequilibrium (LD) adjacent to *MUC5B*, nor did we discover other likely risk-contributing variants elsewhere in the genome. Whole genome sequencing thus corroborates the association of rs35705950 with *MUC5B* dysregulation and interstitial lung disease. This novel exercise additionally served a unique mission in bridging clinical and basic science education.

## Introduction

Recently, several studies have supported the value of clinical genome sequencing, particularly when there is diagnostic uncertainty [1]–[4]. As clinical genome sequencing becomes more widely available, it is likely to provide useful information, even when there is no family history of disease. However, interpretation of genomic data has not yet been widely incorporated into medical school curricula, and how such studies can best be employed to inform medical practice remains a subject of intense interest [5].

To address emerging implications for applying clinical genomics, students earning concurrent M.D. and Ph.D. degrees in the University of Washington Medical Scientist Training Program (MSTP) participate in a new course, also open to a limited number of M.D.-only students, in which a cadaver undergoes whole genome sequencing in association with dissection in the human anatomy lab. The cadaver selected for the inaugural exercise had been an otherwise healthy male with non-familial idiopathic pulmonary fibrosis (IPF).

Interstitial lung diseases can be difficult to clinically and pathologically characterize [6]. Many cases are diagnosed as idiopathic, highlighting a need to develop better understanding of their pathogenesis. Current evidence suggests that IPF follows a “two-hit” disease model where hereditary factors alter underlying disease susceptibility to environmental stressors such as cigarette smoke, asbestos, or silica [7], [8]. Familial IPF kindred and genome-wide association studies (GWAS) have identified several genetic variants that alter disease susceptibility [9]–[15]. Interestingly, many of the variants described in the literature are known to be involved in host defense, cell adhesion, maintenance of genomic integrity, and preservation of lung architecture. To cite one prominent example, a single nucleotide polymorphism (SNP) in the promoter of the *MUC5B* gene was recently reported to up-regulate expression of the gene and is both genetically linked and associated with IPF [10]–[14], [16].

As an exercise in medical education, we interpret the cadaver's genome sequence in concert with gross and microscopic anatomical examination and discuss its potential relevance for diagnosis and management of IPF.

## Materials and Methods

### Ethics

Written informed consent for body donation for research and education was obtained through the University of Washington School of Medicine Willed Body Program. All research was conducting according to Declaration of Helsinki principles.

### Genome sequencing

Tissue samples were obtained postmortem from the unembalmed cadaver. A cube of liver tissue approximately 1 cm on a side was dissected from the liver and frozen at −80°C to be used in the preparation of DNA for sequencing. 17 µg of genomic DNA was extracted using Qiagen DNeasy Blood & Tissue Kit from this sample. Shotgun sequencing libraries were prepared using the KAPA library preparation kit (Kapa Biosystems) following manufacturer's instructions. DNA was sequenced on an Illumina HiSeq 2000 with paired-end 100 bp reads. The raw sequence data was mapped to hg19/GRCh37 and variants were called with GATK using best practices [17]–[19]. Variants were annotated using SeattleSeq [20]. Allele frequencies for the European-American population were derived from the University of Washington Genome Variation Server (http://gvs.gs.washington.edu). Findings discussed in the manuscript were manually evaluated by verifying read data using the Integrative Genomics Viewer (IGV, http://www.broadinstitute.org/igv/).

### Analysis of variants in the vicinity of rs35705950 (*MUC5B*)

To assess the possibility of causal variants underlying the *MUC5B* GWAS signal in LD with rs35705950, we used the recently described CADD method [21] to generate “C-scores” for each of the patient's variants within 1 Mb of rs35705950 (Chr11∶1,241,221). LD data from the SNP Annotation and Proxy Search (SNAP) [22] did not identify any variants in the CEU population in high LD (r-squared >0.6) with rs35705950 within 1 Mb. Nonetheless, we ranked variants by C-scores, which are an integrated measure of “deleteriousness” outputted on a “phred-like” scale from 0 to 99 [21]. We additionally investigated a region within 20 kB of rs35705950 to identify any rare (AF<2%) variants that overlapped with DNase hypersensitive or putative transcription factor binding sites.

### Didactics

A group of 15 first year medical students, including 12 combined M.D./Ph.D. students from the Medical Scientist Training Program (MSTP), plus a more senior MSTP student (AK) who functioned as a teaching assistant, met in the Human Anatomy Lab and classroom for a total of 8 hours dispersed through 5 sessions. In smaller groups, the students, under the supervision of the teaching assistant, prepared DNA samples for genomic sequencing. Students were then paired off to complete remaining bioinformatic analysis and jointly draft the manuscript using a shared document online over the ensuing academic quarter. Instructors and students met as a group in 3 additional sessions spanning a total of 5 hours in order to refine and revise the manuscript.

## Results

### Clinical history

The patient was a 61 year-old man of European-American ancestry without significant prior medical history who enjoyed good health until approximately eight months before he expired, when he developed flu-like symptoms marked by progressive dry cough and dyspnea. He first sought medical attention when, two months later, he presented with lower extremity edema. Pulmonary artery catheterization at that time revealed severe pulmonary artery hypertension and other changes consistent with *cor pulmonale*. Subsequently, he developed atrial flutter requiring cardioversion and increasing dependence on supplemental oxygen. X-ray computed tomography (CT) of the chest revealed changes consistent with chronic pulmonary fibrosis, comprised of severe dilation of the main pulmonary artery, diffuse basilar ground-glass opacities, and subpleural reticular opacities. The patient had no known family history of pulmonary diseases. He was married and a father. He worked as a truck driver and was previously employed in a chemical manufacturing facility. There was no known history of asbestos exposure. He kept a pet bird as a younger adult. He had not resided in regions associated with endemic fungal disease. Substance abuse was confined to a 32 pack-year history of smoking.

The patient's symptoms worsened despite treatment consisting of diuretics, corticosteroids, and sildenafil. Approximately two months prior to death, he was transferred to our institution for evaluation for lung transplant. Imaging studies and routine clinical laboratory analysis revealed no evidence for thromboembolism, infection, connective tissue disease, or underlying immunodeficiency. During this interval, it became increasingly difficult to maintain adequate oxygenation. Radiographic chest imaging showed progressive lobar consolidation. As hypoxia worsened, mental status deteriorated. His family requested that care be limited to comfort measures, and he died shortly afterward. Lung tissue was not obtained for diagnosis prior to death.

### Gross anatomic findings

The lungs exhibited smooth pleural surfaces, and sectioning revealed diffuse consolidation, without evidence of accentuated subpleural fibrosis and honeycomb patterns of airspace enlargement. The pulmonary vessels showed focal intimal thickening and plaque formation, consistent with pulmonary hypertension, but no evidence of recent or remote thromboemboli. The hilum and mediastinum contained enlarged, reactive-appearing lymph nodes. The heart was also enlarged, and all chambers were hypertrophic. The right ventricle demonstrated marked muscular hypertrophy, and had a wall thickness that equaled the left ventricle, consistent with *cor pulmonale*. There was no significant atherosclerotic coronary artery disease, valvular disease, or evidence of myocardial infarction.

### Microscopic findings

**Figure 1:**
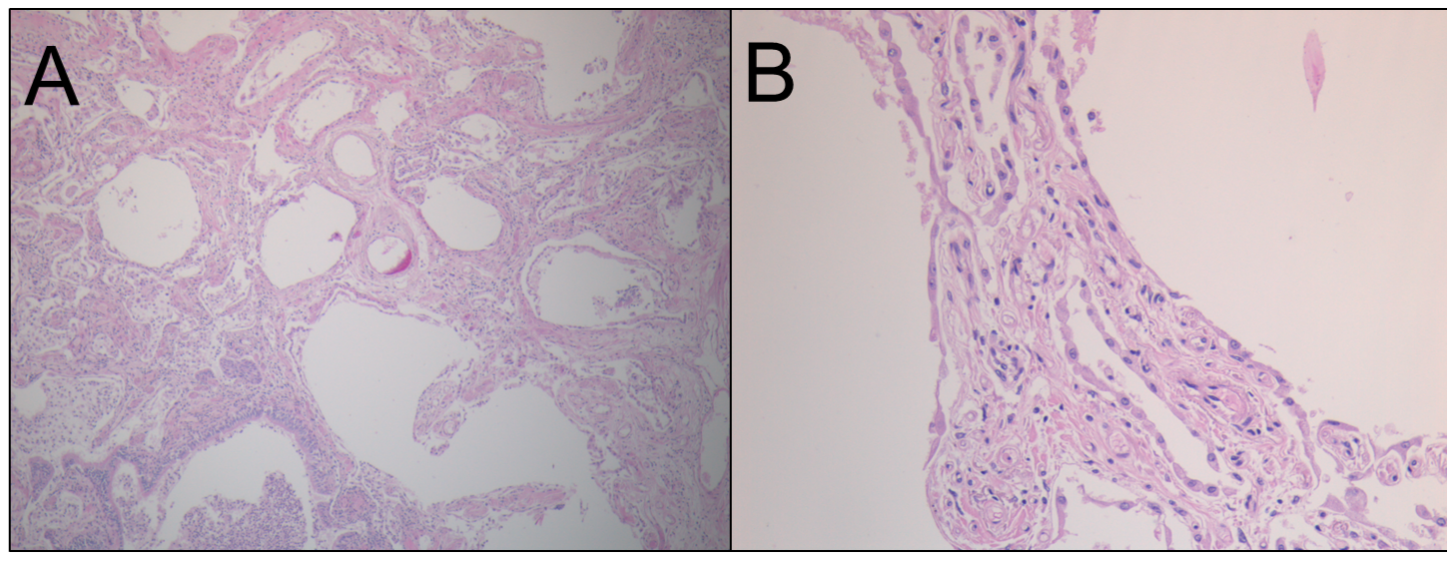
Histological demonstration of the NSIP pattern of IPF in the patient's lungs. Microscopic examination of the lungs. (**A**) 40×; (**B**) 400×. Note uniform fibrotic thickening of the alveolar septae and type II pneumocyte hypertrophy. There was no histologic evidence of sarcoidosis, hypersensitivity pneumonitis, organizing pneumonia, or diffuse alveolar damage.

Histologic examination of lung tissue revealed diffuse fibrous thickening of alveolar septae (Figure 1, Figure S1). Changes were relatively uniform throughout the lung. Dense bundles of collagen and scant mononuclear inflammatory cell infiltrates existed within thickened septae. Focally, apical subpleural regions exhibited increased fibrosis and remodeling, with associated airspace enlargement. To further characterize the histologic changes and classify this patient's pulmonary fibrosis, we processed an additional 10 blocks of tissue from representative sections of all lobes of the right and left lung. The pattern of injury was uniform throughout the lungs, showing diffuse fibrocellular thickening of the vast majority of alveolar septae. One section from the right middle lobe contained changes of microscopic honeycombing in an area of subpleural fibrosis, where small cysts lined by respiratory epithelium are present and contain mucous and neutrophils (Figure S1). Microscopic honeycombing has been reported in NSIP and other lung diseases, and to our knowledge this entity is not specific for UIP [23], [24]. The temporal uniformity of the process, and clinical presentation are most compatible with a pathologic diagnosis of the fibrosing variant of nonspecific interstitial pneumonia (NSIP), in contrast to the initial diagnosis of IPF [25]. Some of the pulmonary arteries demonstrated fibrous intimal thickening, and the myocardium showed myocyte hypertrophy. Alveolar hemosiderin-laden macrophages were present within lung sections and most likely reflect pulmonary hemorrhage secondary to pulmonary hypertension. Finally, pathological sections revealed an acute bronchopneumonia, consistent with terminal bronchopneumonia most likely due to aspiration. Lymph nodes showed only nonspecific reactive changes.

### Genome sequencing

We performed whole genome sequencing, yielding 1.14 billion read pairs resulting in 52× median coverage across the mappable genome (3.1 Gb) and 44× median coverage across the exome (36.6 Mb). A total of 3.2 million single nucleotide variants (SNVs) were identified across the genome, with a Ti:Tv of 2.12 [19]. A total of 29,646 SNVs altering protein-coding were observed, of which 146 were not seen in dbSNP v137. Additionally, 305 novel coding indels not reported in dbSNP v137, were also identified.

### Mendelian disorders

Mutations in several genes have been reported to segregate with familial forms of IPF (Table 1). We searched the patient's genome for rare variants (minor allele frequency (MAF)<0.01) in protein-coding regions within this set of genes. We did not identify rare variants predicted to alter protein sequence or splicing in these genes, although we did find 3 rare synonymous codon substitutions in each of *TERT*, encoding a component of telomerase; *DSP*, producing desmoplakin, a component of desmosomes; and *DPP9*, the product of which is a serine protease. While synonymous changes in protein coding regions are increasingly reported to influence heritable susceptibility to disease [26], the significance of these variants remains uncertain. DNA sequence analysis software also detected coding variants in *MUC2*, but upon further scrutiny we interpreted them as artifacts attributable to DNA alignment errors due to sequence similarity among mucin gene family members [13], [27].

**Table 1:** Genes previously associated with familial IPF.

Gene	Mutation	Reference
*ABCA3*	c.839G>A/p.R280H	[40]
	c.863G>A/p.R288K	[40]
	c.2891G>A/p.G964D	[41]
	c.8784A>G/p.S1262G	[40]
*MICA*	Alleles distinguished by multiple variants	[42]
*SFTPA1*	Alleles distinguished by multiple variants	[43]
*SFTPA2*	c.593T>C/p.F198S	[44]
	c.692G>T/p.G231V	[44]
*SFTPC*	c.116T>C/p.V39A	[45]
	c.211A>G/p.M71V	[40]
	c.218T>C/p.I73T	[40], [46], [47]
	c.298G>A/p.G100S	[48]
	c.325-1G>A/IVS3-1	[45]
	c.434+1G>C/IVS4+1	[49]
	c.435+2T>C/IVS4+2	[40]
	c.424delC/p.H142fs	[45]
	c.435G>C/p.Q145H	[45]
	c.563T>C/p.L188P	[45]
	c.563T>A/p.L188Q	[50]
	c.566G>A/p.C189Y	[45]
	c.581T>A/p.L194P	[45]
*TERT*	c.97C>T/P33S	[51]
	c.164T>A/L55Q	[52]
	c.277+1G>A/IVS1+1	[52]
	c.334delC/p.112fs	[52]
	c.430G>A/V144M	[51]
	c.1456C>T/R486C	[51]
	c.1892G>A/p.R631Q	[53]
	c.2240delT/V747fs	[51]
	c.2594G>A/R865H	[51], [53]
	c.2648T>G/p.F883C	[53]
	c.2712-2A>C/IVS9-2	[52]
	c.3329C>T/p.T1110M	[52]
	c.3346_3522del/E1116fs	[51]
*TERC*	r.98g>a	[52]
	r.37a>g	[51]
	r.52_86del (also described as r.53_87del)	[54]

### GWAS variants

**Figure 2:**
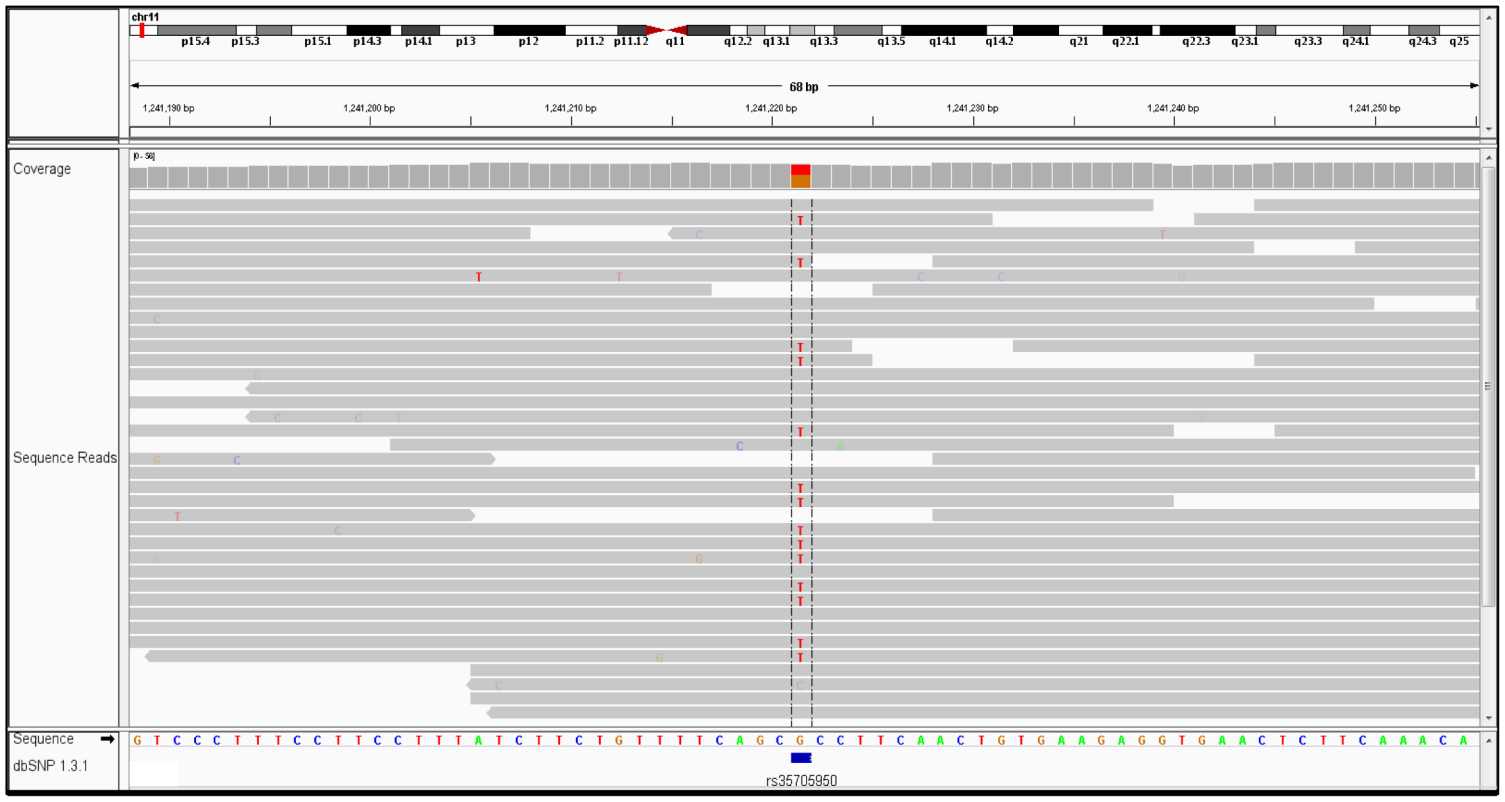
Integrated Genomics Viewer (IGV) screenshot of the rs35705950 variant.

We next cross-referenced the patient's genome with SNPs previously implicated in IPF by GWAS (Table S1). The patient was heterozygous for six variants influencing susceptibility to IPF (Table 2). Two of the six are associated with elevated risk for IPF. One of these variants (Figure 2), rs35705950, is located within the promoter region of *MUC5B* and has a strong association with both familial and sporadic IPF, with odds ratio (OR) estimates ranging from 2.4–6.8 for heterozygote carriers. The presence of one other variant located on chromosome 7 also increases risk for IPF [13]. Four of the six variants are associated with reduced susceptibility to IPF and include SNPs in *OBFC1*, a gene involved in telomere maintenance, *MAPT*, the gene from which the microtubule-associated protein tau is produced [13], as well as the Toll interacting protein, TOLLIP, and signal peptidase, SPPL2C [10].

**Table 2:** Variants associated with IPF that were also seen in this individual. Allele frequencies accessed 4/16/2014.

Nearest Gene	SNP ID	Chromosome	Position	Variant Type	Minor Allele	Major Allele	Patient Genotype	MAF (1000 Genomes)	OR	Reference
*AZGP1* (*AZGP1P1* pseudogene)	rs4727443	7	99593346	intergenic	A	C	C/A	0.411	1.3^A^ 1.11^B^	[13]
*MAPT*	rs1981997	17	44056767	intronic	A	G	A/G	0.117	0.71^A^ 0.67^B^	[13]
*MUC5B*	rs35705950	11	1241221	promoter	T	G	T/G	0.052	2.4–6.8	[10]–[14], [16]
*OBFC1*	rs11191865	10	105672842	intronic	G	A	A/G	0.584	0.8^A^ 0.87^B^	[13]
*TOLLIP*	rs5743890	11	1325829	Intronic	C	T	C/T	0.0702	0.61	[10]
*SPPL2C*	rs17690703	17	43925297	Intronic	T	C	C/T	0.1543	0.7	[10]

A Discovery and ^B^replicate GWAS.

### Analysis of variants in the vicinity of rs35705950 (*MUC5B*)

**Figure 3:**
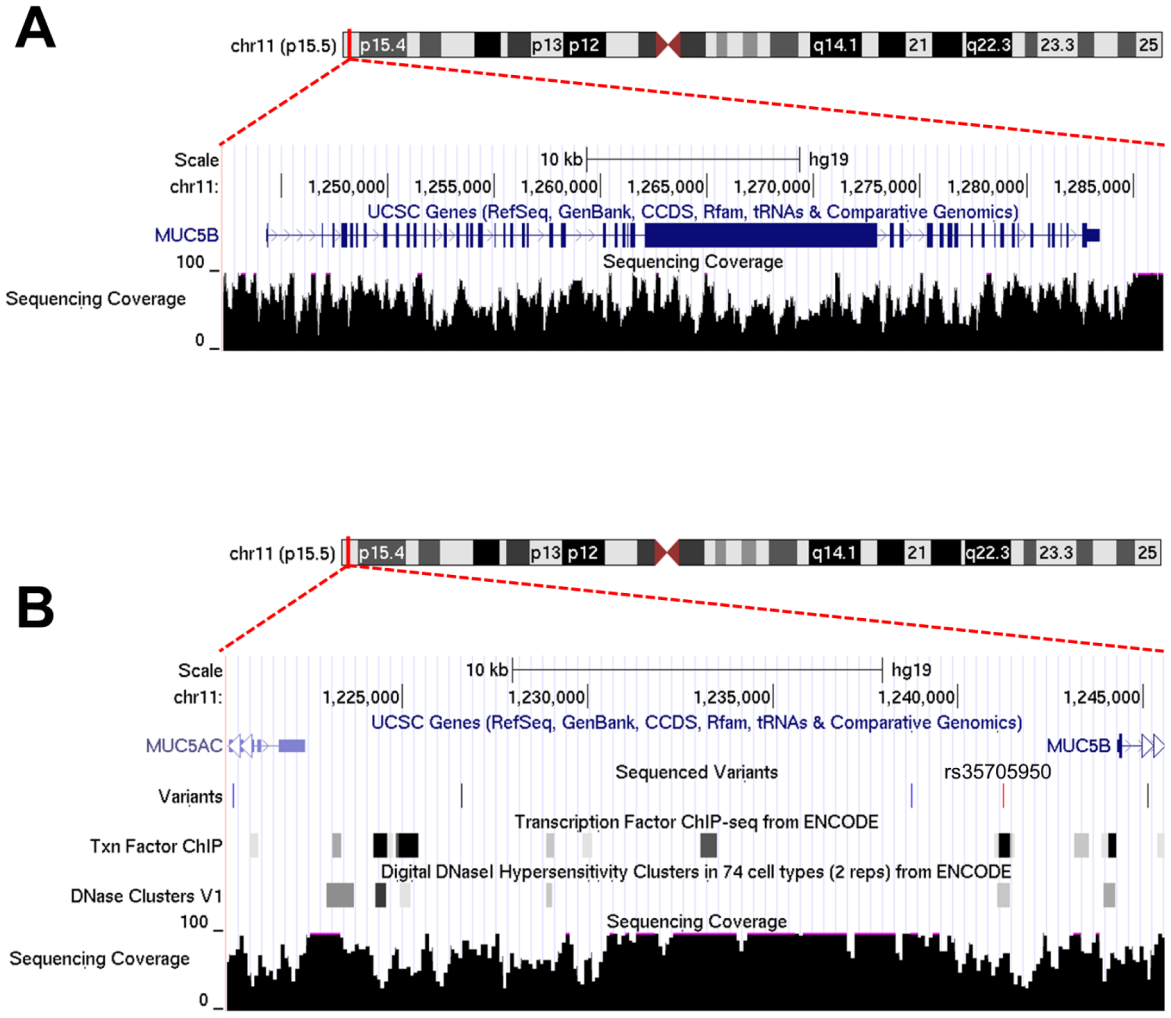
Sequencing coverage and variant distribution within the *MUC5B* locus. (**A**) Sequence coverage of the *MUC5B* gene. (**B**) Rare variants neighboring the *MUC5B* promoter variant rs35705950. Variant track is colored by allele frequency (blue: AF<2%, red: AF<5%, black: AF<10%). No other rare variants in this region overlap with putative transcription factor binding sites, consistent with the hypothesis that the rs35705950 is causative of *MUC5B* dysregulation. Plots were generated using the UCSC genome browser (http://www.genome.ucsc.edu).

The availability of whole genome sequence allowed us to explore whether the *MUC5B* promoter variant contributes to increased risk for IPF, as opposed to alternatively serving only as a marker in linkage disequilibrium (LD) with other causative variant(s) in the same region (Figure 3a, Figure S2). For this analysis we used a recently described approach, Combined Annotation-Dependent Depletion scoring system (CADD) [21], which estimates the relative pathogenicity of variants based on a variety of predicted functional effects. We first used HapMap data for individuals of European ancestry to define LD in the vicinity (1 Mb in either direction) of rs35705950. The highest scoring SNV out of 2,284 in the region, a nonsense variant within *MUC6* (C-score  = 42) failed manual validation, due to likely misalignment, and no other SNVs had C-scores above 23. The full table of variants and scores is provided as Table S2. We also investigated the region between the *MUC5AC* gene and the *MUC5B* start site for rare variants (AF<2%) that overlap with regions that may possibly influence expression (Figure 3b). Only one SNP, rs35705950, overlapped with both DNAse hypersensitivity and transcription factor binding regions.

### Rare coding variants

To assess genes not previously associated with IPF but potentially relevant to observed pathology, we filtered variants for rare protein-altering SNVs. With a MAF threshold of 0.01, we identified 1,291 novel or rare coding SNVs. A subset of 57 coding variants at nucleotide positions demonstrating significant interspecies conservation (conScoreGERP >5.75) were delineated (Table S3). GeneCards (http://genecards.org) and literature searches were consulted to determine the function, associated disease, and expression profile of each of these variants. Few of these variants are known to be expressed in lung or specifically in diseased lung tissue from patients with IPF [28]; however, this does not rule out their contribution to disease as the expression profile may be incomplete, the gene's effect on the lung may be indirectly mediated through exogenous inflammatory pathways, or the deleterious effects of the genes may arise from abnormal expression. We compared our list of novel variants to a previously published set of genes that are differentially expressed between IPF patients and controls [28]. Twenty-nine/146 of the novel variants are included in this set. Three are nonsense variants: *NCKAP5* and *SLC25A25*, which were underexpressed in IPF patients, and *MNS1/TEX9*, which was overexpressed in IPF patients. Other coding variants for genes in this list have previously been associated with different inherited disorders, but none seem pertinent to the patient's illness.

## Discussion

We present what we believe to be the first human genome sequence performed on an individual carrying a clinical diagnosis of IPF. His otherwise excellent health affords a unique opportunity to uncover genetic factors specifically contributing to development of pulmonary disease.

Infrequently, mutations in several genes are linked to heritable, highly-penetrant forms of IPF. The patient lacked rare coding sequence alterations in any of the previously identified genes, in accord with an absence of a family history of pulmonary disease.

Nevertheless, common heritable variants, in this case not altering protein coding, have been found through linkage analysis and GWAS to contribute to risk for IPF. For those for which published evidence is most robust, the patient had a mixture of both risk-reducing (rs1981997, rs11191865, rs5743890 and rs17690703) and risk-elevating (rs4727443 and rs35705950) alleles [11], [13]. Among them, rs35705950, a SNP contained in the promoter of *MUC5B*, outweighs any of the others in markedly predisposing to development of IPF (OR, 2.4–6.8, for heterozygous carriers) [11].

*MUC5B* encodes for mucin 5B glycoprotein, which is expressed in saliva and lung tissue and is thought to have lubricating and viscoelastic properties [29]. Recently it has been shown to play an important role in mucociliary clearance, defense against pulmonary infection, and regulating airway inflammation [30].

A tissue diagnosis was not made during the patient's life. Microscopic analysis of tissue obtained upon gross dissection indicates that the patient's pulmonary disease is more appropriately classified as NSIP. In distinction with IPF, NSIP tends to occur at a younger age, is associated with a better clinical outcome, and occurs in a wide variety of clinical contexts, sometimes in association with an underlying disorder [25]. However, by history and clinical laboratory examination, a predisposing disorder remains undiscovered. One exception, though, was the patient's extensive smoking history, which is a known risk factor for IPF [31], though only indirectly so for NSIP [32].

It is worth noting that the *MUC5B* variant, while initially detected in genetic studies exclusively investigating IPF, has also been associated with similarly appearing fibrotic lung disease detectable by chest CT imaging [12]. Given a paucity of other risk factors, it seems reasonable to hypothesize that the *MUC5B* variant contributed to development of NSIP in this patient although further studies are certainly required to explore this link.

We also believe that this is the first whole genome sequence completed on an individual with the *MUC5B* variant. We are therefore in a position to address, first, whether rs35705950 is merely in LD with other adjacent variants that may actually be disease-causing and, second, whether variants at other loci modulate the risk for fibrotic lung diseases associated with this SNP.

With respect to the first question, rs35705950 is located within the promoter region of *MUC5B*, is predicted to disrupt transcription factor binding sites, and is correlated with elevated MUC5B expression [11]. Nevertheless, in contrast to whole genome sequencing, not all variants residing on a common haplotype have necessarily been identified and tested for association with disease. We therefore searched contiguous DNA sequence for additional adjacent variants in the vicinity of rs35705950, but did not find additional variants in apparent LD with rs35705950 that either fell within a known DNase hypersensitive site, transcription factor binding site, or were otherwise predicted to be deleterious. Thus, our data do not detract from the hypothesis that rs35705950 is causative of MUC5B dysregulation and disease association.

Relevant to the second question, we searched for all rare and novel variants in the patient's genome, including those not previously associated with IPF. There were no immediately plausible candidates amongst the hundreds of genes for which the patient, as would be expected for anyone [33], harbored rare and private variants. However, nonsense variants were found in the peripheral clock gene *NCKAP5* and the calcium-binding mitochondrial carrier *SLC25A25*, which are each down-regulated in IPF [28].

The patient's somewhat acute presentation following onset of flu-like symptoms is perhaps consistent with an antecedent viral infection, which is a setting in which NSIP has been known to occur [34]. In principle, genomic sequence analysis could permit identification of pathogens that might have triggered putative immune responses and ultimately set the stage for development of fibrotic lung disease. The DNA sequence read mapping strategy we pursued here, involving alignment to a reference genome, filters away the DNA sequences of other organisms. Moreover, for reason of convenience, DNA was extracted from liver, prior to embalming, whereas DNA extraction from lung tissue or regional lymph nodes would have served better for the purpose of detecting the genomes of pathogens.

In addition to offering insight into the pathogenesis of the patient's lung disease, whole genome sequence information may also help to infer prognosis and guide treatment. For example, although this patient unfortunately suffered a rapidly progressive course, in general, the presence of the *MUC5B* SNP rs35705950 has been recently shown to confer a more favorable prognosis [27]. Other potentially identifiable genetic variants are associated with habitual tobacco use and may be used to help guide smoking cessation strategies [35], thus mitigating at least one controllable risk factor for lung disease. Although, again unfortunately, this patient did not survive to lung transplant, and while there are alternative conventional serological approaches available for tissue typing, whole genome sequence information [36] can be used to precisely match blood types, which is strongly preferred for lung transplant [37], as well as refine HLA typing, which, when matched between donor and recipient, improves outcomes [38].

Needless to say, genomic sequence is useful for genetic counseling and providing risk assessment to relatives. In this particular situation—body donation for education and research—there is no intent to communicate findings to loved ones. Nevertheless, post-mortem genomic analysis could conceivably enhance the educational and research value of autopsy, as well as return risk predictive information to survivors.

Finally, we wish to comment on the didactic value of this exercise, which, in addition to its research contribution, fulfilled several educational objectives. Medical history of the cadaver is not typically available in human anatomy courses [39]. In this case, the students participating in the course received the benefit of a case presentation from a physician who had cared for the patient while hospitalized. It is also unusual to combine the goals of an autopsy with gross dissection [39]. Similarly, histopathologic examination of embalmed tissue is typically not also performed on cadavers in human anatomy courses [39]. In this course, both the gross and microscopic examination of tissues was performed under the guidance of a clinical pathologist, offering a unique opportunity to tie together clinical, anatomic, and cellular findings. Students collectively performed the laboratory and bioinformatic analysis required to assemble the patient's genome and jointly interpreted genetic findings employing literature searches, as well as a variety of databases and computational approaches. Students collectively drafted the manuscript. In summary, this novel case study promoted teamwork and honed clinical, laboratory, computational, writing, and other skills important for career development of physicians and scientists, while contributing genetic insight into a poorly understood disease.

## Supporting Information

Figure S1 **Additional gross/histologic features suggestive of NSIP.** A. Gross view. B. Histology suggestive of NSIP, with diffuse fibrocellular thickening of alveolar septae (40× Magnification). C. Microscopic honeycombing found in right middle lobe (40× Magnification). Small cysts lined by respiratory epithelium are present.
(PDF)

Figure S2 **Sequence coverage across the mucin cluster at chromosome 11p15.5.** Sequence coverage of this individual is shown up to 100-fold coverage. A gap in sequence coverage from position 1,160,000 to 1,213,000 is actually the result of missing sequence in the human reference assembly (shown in orange).
(PDF)

Table S1 **IPF-associated SNPs investigated in this study.** MAF accessed 1/20/2014. Includes data from Table 2. ^A^Discovery and ^B^replicate GWAS.
(PDF)

Table S2 **CADD analysis to estimate the relative pathogenicity of variants in the vicinity of rs35705950.** File is in Microsoft Excel.xlsx format.
(XLSX)

Table S3 **Rare/novel protein-altering variants.** Coding variants with a GERP score >5.75 and MAF<0.01 (as determined through the ESP server) are shown here. Genes with multiple variants (*OR4C3, LOC100996481*) are likely artifacts resulting from improperly aligned sequence reads.
(PDF)
