# Supplementary material for: Genome Sequencing of Idiopathic Pulmonary Fibrosis in Conjunction with a Medical School Human Anatomy Course
Source: PLoS One. 2014 Sep 5;9(9):e106744. doi: 10.1371/journal.pone.0106744 (PMC4156421; doi:10.1371/journal.pone.0106744)
Supplement: Figure S1 — Additional gross/histologic features suggestive of NSIP. A. Gross view. B. Histology suggestive of NSIP, with diffuse fibrocellular thickening of alveolar septae (40× Magnification). C. Microscopic honeycombing found in right middle lobe (40× Magnification). Small cysts lined by respiratory epithelium are present. (PDF) [file pone.0106744.s001.pdf]

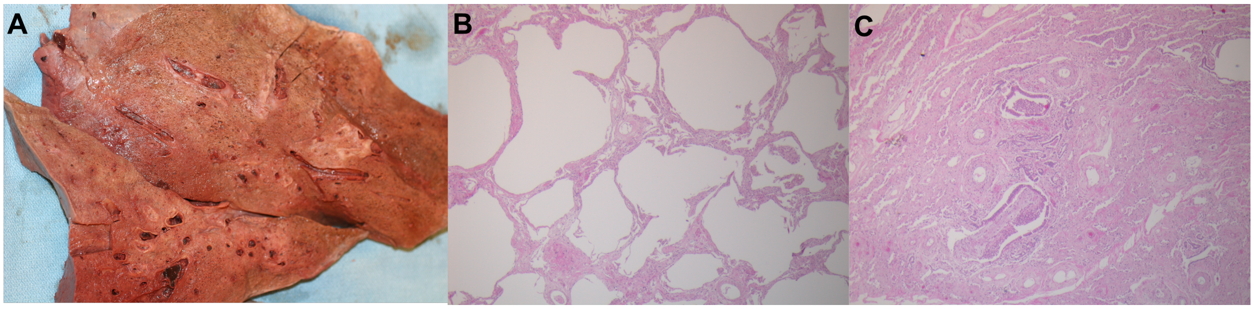

Figure S1. Additional gross/histologic features suggestive of NSIP. A. Gross view. B. Histology suggestive of NSIP, with diffuse fibrocellular thickening of alveolar septae (40x Magnification). C. Microscopic honeycombing found in right middle lobe (40x Magnification). Small cysts lined by respiratory epithelium are present.
