# Supplementary material for: Genome Sequencing of Idiopathic Pulmonary Fibrosis in Conjunction with a Medical School Human Anatomy Course
Source: PLoS One. 2014 Sep 5;9(9):e106744. doi: 10.1371/journal.pone.0106744 (PMC4156421; doi:10.1371/journal.pone.0106744)
Supplement: Figure S2 — Sequence coverage across the mucin cluster at chromosome 11p15.5. Sequence coverage of this individual is shown up to 100-fold coverage. A gap in sequence coverage from position 1,160,000 to 1,213,000 is actually the result of missing sequence in the human reference assembly (shown in orange). (PDF) [file pone.0106744.s002.pdf]

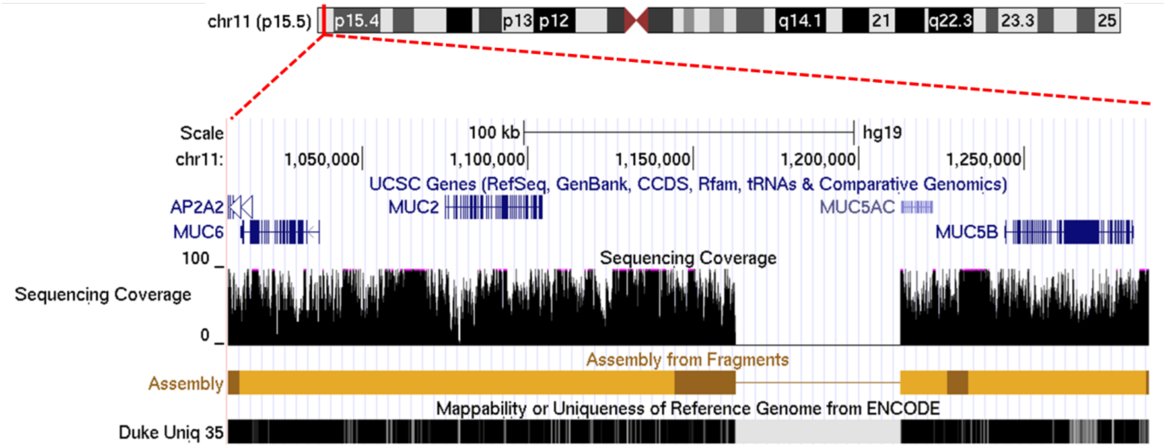

Figure S2. Sequence coverage across the mucin cluster at chromosome 11p15.5. Sequence coverage of this individual is shown up to 100-fold coverage. A gap in sequence coverage from position 1,160,000 to 1,213,000 is actually the result of missing sequence in the human reference assembly (shown in orange).
