# Supplementary material for: Genome Sequencing of Idiopathic Pulmonary Fibrosis in Conjunction with a Medical School Human Anatomy Course
Source: PLoS One. 2014 Sep 5;9(9):e106744. doi: 10.1371/journal.pone.0106744 (PMC4156421; doi:10.1371/journal.pone.0106744)
Supplement: Table S1 — IPF-associated SNPs investigated in this study. MAF accessed 1/20/2014. Includes data from Table 2. ADiscovery and Breplicate GWAS. (PDF) [file pone.0106744.s003.pdf]

Table S1. IPF-associated SNPs investigated in this study. MAF accessed 1/20/2014. Includes data from Table 2. [A]Discovery and [B]replicate GWAS.

Nearest Gene	SNP ID	Chr	Position	Variant Type	Minor Allele	Major Allele	Patient Genotype	MAF (1000 Genomes)	OR	Reference
MUC5B	rs35705950	11	1241221	promoter	T	G	T/G	0.052	6.8 [9]<br>6.3 [10]<br>4.51[11]	[12-14]
AZGP1P1	rs4727443	7	99593346	intergenic	A	C	C/A	0.411	1.3[A]<br>1.11[B]	[14]
MAPT	rs1981997	17	44056767	intronic	A	G	A/G	0.117	0.71[A]<br>0.67[B]	[14]
OBFC1	rs11191865	10	105672842	intronic	G	A	A/G	0.584	0.8[A]<br>0.87[B]	[14]
TERC	rs1881984	3	169464459	intergenic	G	A	G/A	0.327		[14]
LRRC34	rs6793295	3	169518455	Missense	C	T	T/T	0.4261	1.30[A]<br>1.39[B]	[14]
FAM13A	rs2609255	4	89811195	Intronic	G	T	T/T	0.309	1.2[A]<br>1.43[B]	[14]
TERT	rs2736100	5	1286516	Intronic	C	A	C/C	0.4477	0.73[A]<br>0.74[B]	[14,42]
DSP	rs2076295	6	7563232	Intronic	T	**G**	G/G	0.4536	1.43[A]<br>1.26	[14]
MUC2	rs7934606	11	1093945	Intronic	C	**T**	T/T	0.205	1.52[A]<br>1.56[B]	[14]
ATP11A	rs1278769	13	113536627	3' UTR	A	G	G/G	0.2351	0.79[A]<br>0.80[B]	[14]
IVD	rs2034650	15	40717302	Intronic	G	A	A/A	0.4839	0.77[A]<br>0.82[B]	[14]
DPP9	rs12610495	19	4717672	Intronic	G	A	A/A	0.2057	1.29[A]<br>1.30[B]	[14]
TOLLIP	rs111521887	11	1312706	Intronic	G	C	C/C	0.0845	1.48	[11]
TOLLIP	rs5743894	11	1324772	Intronic	C	T	T/T	0.0868	1.49	[11]
TOLLIP	rs5743890	11	1325829	Intronic	C	T	C/T	0.0702	0.61	[11]
SPPL2C/MAPT-AS1	rs17690703	17	43925297	Intronic	T	C	C/T	0.1543	0.7	[11]
TLR3	rs3775291	4	187004074	Missense	T	C	C/T	0.25	N/A from a murine study of IPF	[43]

References refer to main text, with addition of the following:

42. Mushiroda T, et al. A genome-wide association study identifies an association of a common variant in TERT with susceptibility to idiopathic pulmonary fibrosis. *J Med Genet.* 2008;45(10):654-656.

43. O'Dwyer DN, Armstrong ME, Trujillo G, Cooke G, Keane MP, et al. The Toll-like receptor 3 L412F polymorphism and disease progression in idiopathic pulmonary fibrosis. Am J Respir Crit Care Med. 2013;188(12):1442-1450.
