# Supplementary material for: Genome Sequencing of Idiopathic Pulmonary Fibrosis in Conjunction with a Medical School Human Anatomy Course
Source: PLoS One. 2014 Sep 5;9(9):e106744. doi: 10.1371/journal.pone.0106744 (PMC4156421; doi:10.1371/journal.pone.0106744)
Supplement: Table S3 — Rare/novel protein-altering variants. Coding variants with a GERP score >5.75 and MAF<0.01 (as determined through the ESP server) are shown here. Genes with multiple variants (OR4C3, LOC100996481) are likely artifacts resulting from improperly aligned sequence reads. (PDF) [file pone.0106744.s005.pdf]

Table S3. Rare/novel protein-altering variants. Coding variants with a GERP score > 5.75 and MAF < 0.01 (as determined through the ESP server) are shown here. Genes with multiple variants (*OR4C3, LOC100996481*) are likely artifacts resulting from improperly aligned sequence reads.

Chromosome	Position	Nearest Gene	Type	GERP score	SNP ID (if applicable)	MAF (ESP)
1	11884555	*CLCN6*	missense	5.79	198400	8.46E-04
1	22846709	*ZBTB40*	missense	5.79	209720	3.92E-03
1	84948653	*RPF1*	missense	5.93	150021810	1.69E-03
1	120263839	*PHGDH*	missense	5.92	NA	0
2	32379530	*SPAST*	missense	5.76	NA	0
2	33567971	*LTBP1*	missense	5.91	61751742	7.23E-03
2	160136337	*WDSUB1*	missense	5.79	174264	7.69E-05
2	179417175	*TTN*	missense	5.76	NA	0
2	209184980	*PIKFYVE*	missense	5.83	10932258	7.85E-03
2	216272900	*FN1*	missense	5.93	2577301	7.69E-05
3	24006477	*NR1D2*	missense	5.78	4858097	0
3	188592145	*LPP*	missense	5.79	9830664	2.08E-03
4	79458320	*FRAS1*	missense	5.93	199510509	1.33E-03
5	115813726	*SEMA6A*	missense	5.98	34966	0
6	57398270	*LOC100996481*	stop-gained	5.79	62398999	0
6	57467084	*LOC100996481*	missense	5.86	9885913	0
6	57512476	*LOC100996481*	missense	5.94	4307164	0
6	112508770	*LAMA4*	missense	5.9	9400522	0
6	136599393	*BCLAF1*	missense	5.97	6940018	3.61E-03
7	86468254	*GRM3*	missense	5.91	17161026	9.53E-03
7	121682694	*PTPRZ1*	missense	5.86	61757819	2.15E-03
8	52733128	*PCMTD1*	missense	5.96	62506083	0
8	52733231	*PCMTD1*	stop-gained	5.77	75748152	0
9	101804366	*COL15A1*	missense	5.92	35901514	9.15E-03
9	130868065	*SLC25A25*	stop-gained	5.77	NA	0
10	13166076	*OPTN*	missense	6.17	523747	9.38E-03
10	33552696	*NRP1*	missense	5.76	7079053	5.38E-04
10	85972932	*CDHR1*	missense	5.93	137876961	3.46E-03
10	86131370	*FAM190B*	missense	6.17	NA	7.69E-05
11	34152939	*NAT10*	missense	5.76	2957516	2.54E-03
11	48346791	*OR4C3*	missense	5.88	73463994	0
11	48346932	*OR4C3*	missense	5.78	80285195	0
11	48346962	*OR4C3*	missense	5.78	74589050	0
11	48347014	*OR4C3*	stop-gained	5.78	72473368	0
11	48347067	*OR4C3*	missense	5.78	73464001	0
11	48347306	*OR4C3*	missense	5.97	73465911	0
11	67957518	*SUV420H1*	missense	5.77	2512606	0

11	92533558	*FAT3*	missense	5.95	200944979	8.06E-04
11	108380335	*EXPH5*	missense	5.8	1943382	6.15E-04
11	110035240	*ZC3H12C*	missense	5.85	NA	1.64E-04
12	42512830	*GXYLT1*	missense	5.77	76740071	0
12	64587633	*C12orf66*	missense	6.07	699638	6.48E-03
12	95602794	*FGD6*	missense	5.77	142845815	9.23E-04
13	47260054	*LRCH1*	missense	5.93	842381	1.85E-03
14	77810133	*TMED8*	missense	5.98	142882254	2.69E-03
15	48443699	*MYEF2*	missense	5.81	2470103	5.39E-04
15	48807637	*FBN1*	missense	5.93	4775765	0
15	56386577	*RFX7*	missense	5.89	7170589	8.06E-05
16	69725674	*NFAT5*	missense	6.08	NA	0
16	70954915	*HYDIN*	missense	5.89	1798531	6.27E-03
17	15961336	*NCOR1*	missense	5.87	200722329	0
17	40001580	*KLHL10*	missense	6.17	61752339	5.80E-03
17	56544280	*HSF5*	missense	5.81	1017089	2.31E-04
18	29497573	*TRAPPC8*	missense	6.05	6506948	3.84E-04
18	55273151	*NARS*	missense	5.76	79435773	2.46E-03
21	35467645	*SLC5A3,MRPS6*	missense	6.11	8129891	0
22	20082293	*DGCR8*	missense	5.82	35569747	7.00E-03
